# Association between Bone Metabolism and Vestibular Problems in the Modified Romberg Test: Data from the 2009–2010 Korean National Health and Nutrition Examination Survey

**DOI:** 10.3390/jcm9082415

**Published:** 2020-07-28

**Authors:** So Young Kim, Yang-Sun Cho, Ji-Soo Kim, Ja-Won Koo

**Affiliations:** 1 Department of Otorhinolaryngology-Head & Neck Surgery, CHA Bundang Medical Center, CHA University, Seongnam 13496, Korea; sossi81@hanmail.net; 2 Department of Otorhinolaryngology-Head and Neck Surgery, Samsung Medical Center, Sungkyunkwan University School of Medicine, Seoul 06351, Korea; yangsun.cho@gmail.com; 3 Department of Neurology, Seoul National University Bundang Hospital, Seoul National University College of Medicine, Seongnam 13620, Korea; jisookim@snu.ac.kr; 4 Department of Otorhinolaryngology-Head and Neck Surgery, Seoul National University Bundang Hospital, Seoul National University College of Medicine, Seongnam 13620, Korea

**Keywords:** osteoporosis, vitamin D, estrogens, vestibular diseases, cohort studies

## Abstract

Osteoporosis contributes to the occurrence of falling and vestibular problems, particularly in elderly patients. This study aimed to investigate the association between bone metabolism with vestibular problems and falling. A total of 4054 participants of the Korean National Health and Nutrition Examination Survey (KNHANES) from 2009 to 2010 aged ≥50 years old were surveyed on their history of falling, vestibular problems evaluated by the modified Romberg test, variables involving bone metabolism, and serum levels of vitamin D and alkaline phosphatase. They also underwent dual energy X-ray absorptiometry. The crude (simple) and adjusted odd ratios (ORs) of variables involving bone metabolism for vestibular problems in the modified Romberg test and falling were analyzed using a logistic regression model. A subgroup analysis was performed according to sex and the presence of menopause in females. Vestibular problems in the modified Romberg test group but not the falling group were associated with decreased serum vitamin D levels (*p* < 0.001; odds ratio (OR) = 0.951; 95% confidence interval (CI), 0.926–0.976). In subgroup analysis according to sex, the post-menopause group showed a higher rate of vestibular problems in the modified Romberg test compared to the pre-menopause group (4.5% vs. 0.7%, *p* = 0.019). In the post-menopause group, osteoporosis was positively associated with vestibular problems in the modified Romberg test (*p* = 0.001, OR = 10.971, 95% CI = 2.650–45.414). On the other hand, a history of hormone replacement therapy was negatively related with vestibular problems in this subgroup (*p* = 0.035; OR = 0.473; 95% CI = 0.239–0.948). A decrease in serum vitamin D levels may impact the vestibular system through neural signaling or by osteoporotic changes of the otic capsule, as well as otolith particles. Decreased estrogen levels in postmenopausal women may make them more prone to osteoporotic changes, which were associated with vestibular problems in the modified Romberg test. Because this is a cross-sectional study, the causal relationship of bone metabolism with vestibular function needs to be investigated.

## 1. Introduction

The incidence of dizziness and falling is common in the elderly populations [1], and is frequently complicated with other degenerative changes in the cardiovascular and musculoskeletal systems, which makes rehabilitation more difficult [2,3]. Osteoporosis has been reported to be associated with an increased risk of falling and fractures [4,5]. Osteoporosis is a common degenerative disease whose lifetime prevalence was estimated to be approximately 30% in women and 12% in men [6]. Osteoporosis and osteopenia are a spectrum of degenerative bone diseases defined by decreased bone mineral density (BMD) as a result of osteoporotic bone metabolism, including defects in the microarchitecture of bone, poor intrinsic properties of bone, dysfunction of repair of damaged bone, and excessive bone remodeling [6,7]. The fragility of osteoporotic bone and underlying comorbidities could contribute to the risk of falling in the elderly population [5]. In addition, decreased balance has been proposed as the cause for the increased incidence of falling in osteoporotic patients. However, it is difficult to distinguish the contribution of decreased balance from fragility-related falling and other comorbidities, because decreases in balance occur as a consequence of degeneration of the visual, proprioceptive, central nervous, and vestibular systems, or their combination.

Several studies have shown an association between osteoporotic changes and vestibular disease [8,9]. The association of osteoporosis with benign paroxysmal positional vertigo (BPPV) has been described because of degenerative otoconial changes that depend on osteoporotic changes [10]. Decreased BMD, bone turnover markers, and serum vitamin D levels, which are associated with bone metabolism, were correlated with the incidence and recurrence of BPPV [8,9]. In addition to otoconial changes, osteoporotic bone metabolism could lead to vestibular dysfunction via changes in the endolymph and the vestibular bony labyrinth [11]. However, only a few previous studies investigated the relationship of osteoporosis with vestibular dysfunction, although balance-training was found to decrease the risk of falling in patients with osteoporosis [4]. Two cross-sectional studies reported the association of decreased BMD with vestibular dysfunction as measured using the modified Romberg test [9,11]. On the other hand, other clinical studies did not show an association between osteoporosis and balance [12,13]. These contrasting results originate from two main sources. First, the variables leading to osteoporotic changes are diverse and have no standardized reference values. Some variables, such as serum vitamin D and parathyroid hormone (PTH) levels, may change due to other pathological conditions in addition to osteoporosis. Second, differences among study designs are observed, particularly in the demographic of their respective study populations. For example, osteoporosis is more prevalent in the elderly [14] and in postmenopausal women whose bone metabolism is affected by estrogen level [15]. Therefore, these specific susceptibilities and characteristics must be considered in order to elucidate the relationship between bone metabolism and vestibular problems.

There were two hypotheses of the present study. The first hypothesis is that changes in molecules related to osteoporotic bone metabolism, in addition to decreased BMD, could impact vestibular function independent of falling in the middle-aged and old population. In addition, it was also hypothesized that sex differences affected this association due to the effect of estrogen deficiency on osteoporosis in postmenopausal women [16]. To test these hypotheses, we analyzed factors associated with falling and vestibular problems, as well as investigated the risk factors for each disease entity. In particular, we focused on the relationship between osteoporotic changes and vestibular problems. We also analyzed subgroups according to sex.

## 2. Materials and Methods

### 2.1. Study Population and Data Collection

The Korean National Health and Nutrition Examination Survey (KNHANES) is an ongoing cross-sectional survey of the non-institutionalized population of the Korea managed by the Korean government. Every year, 10,000–12,000 subjects from approximately 4600 households are selected from a panel to represent the population, using a multistage clustered and stratified random sampling method based on the national census data.

**Figure 1:**
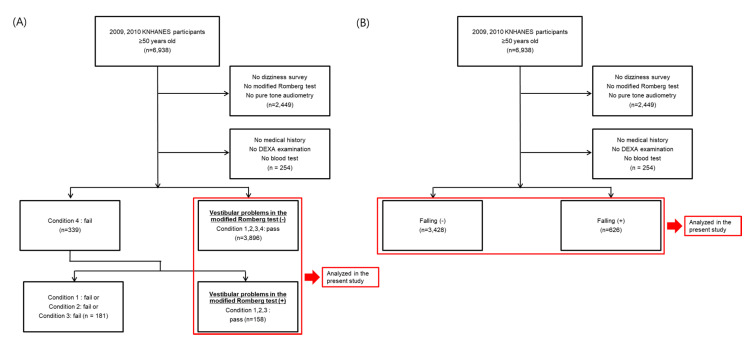
Schematic illustration of the method used for participant selection. (**A**) The participants with vestibular problems in the modified Romberg test were compared with control participants. (**B**) The participants with histories of falling were compared with control participants.

A total of 6938 participants who represented the general Korean population ≥50 years old were enrolled in this study (Figure 1). All enrolled subjects underwent a survey on medical histories as well as a physical examination including a balance function test. A hearing test, blood tests to measure serum vitamin D, PTH, and alkaline phosphatase (ALP) levels, as well as dual energy X-ray absorptiometry (DEXA) were performed as described below.

Written informed consent was obtained from all participants prior to the survey, and approval for this research was given by the Institutional Review Board of the Samsung Medical Center (IRB No. 2013-02-031).

### 2.2. Evaluation of Falling, Modified Romberg Test, and Pure Tone Audiometry

All enrolled participants asked about the any history of falling within the past 12 months without external force (yes or no) and the presence of falling history was defined as the falling (+). The modified Romberg test was performed to evaluate balance function under four conditions, as described previously [17,18]. In brief, participants were positioned to stand with their feet 10 cm apart without bending their knees or moving their bodies to maintain balance on a firm surface, with their eyes open (condition 1) or closed (condition 2). Then, they balanced on an 18-cm-thick, medium-density foam pad (polyurethane, 22 kg/m^3^), with their eyes open (condition 3), or closed (condition 4). The test was passed if the participant keeps the position for >15 s under conditions 1 and 2 and for >20 s under conditions 3 and 4. Participants failed the test if they moved their feet, unfolded their hands, opened their eyes, or needed help maintaining their posture. The participants who failed only condition 4 were considered to have vestibular problems in the modified Romberg test [19,20].

Hearing impairment was evaluated based on the pure tone air-conduction threshold measured in a soundproof booth using an automatic audiometer (GSI SA-203, Entomed Diagnostics AB, Lena Nodin, Sweden). Hearing loss (HL) was defined as >25 dB HL on the average air-conduction hearing thresholds at 0.5, 1, 2, and 3 kHz [21]. The history of tinnitus was also included as a survey item.

### 2.3. Factors Associated with Dizziness and Bone Metabolism

Demographic data on age, sex, and body mass index (BMI) were collected from all participants. BMI (kg/m^2^) was calculated by measuring height and weight and dividing the weight by the height squared.

The medical survey was conducted using previous medical history and physical activity based on the questions (yes or no). Questions about medical history included diagnoses of stroke, osteoarthritis, depression, dyslipidemia, hypertension, diabetes, or anemia by professional clinicians. All survey data were collected by trained staff using a standardized protocol [22]. The psychological condition of the participants was determined by inquiring about depressive mood, limitations in activities due to depression or anxiety, recognition of stress, and quality of life (evaluated using the EuroQol five-dimensional descriptive system (EQ-5D) score from the EuroQol group) [23].

### 2.4. Evaluation of Bone Metabolism and Osteoporosis

Laboratory blood tests included 25-OH vitamin D3, PTH, and ALP levels. 25-OH Vitamin D3 was measured by radioimmunoassay using a gamma counter (Hewlett Packard, Palo Alto, CA, USA). PTH was measured using a LIAISON chemiluminescence immunoassay (Diasorin, Stillwater, MN, USA), and ALP was measured using an enzyme assay using a Hitachi Automatic Analyzer 7600 (Hitachi, Tokyo, Japan) and external and internal quality controls.

Bone mineral density (BMD) (g/cm^2^) was measured by DEXA scan (DISCOVERY-W fan-beam densitometer; Hologic Inc., Bedford, MA, USA). The accuracy of BMD in KNHANES was assessed by educated affiliated osteoporosis assessors, and interpretation of the BMD results was corrected and standardized by quality control [24].

BMD was estimated at lumbar vertebrae (L1–4) and the femur (total femur and femoral neck). The T-score, which is a reference standard deviation value, compared with BMD of a healthy 30-year-old, was used to compare BMD between groups. Osteoporosis was defined when the T-score was ≤ −2.5, and a T-score > −2.5 but < −1 was classified as osteopenia [25].

### 2.5. Statistical Analysis

Statistical analyses were performed using SPSS ver. 18.0 (SPSS Inc., Chicago, IL, USA). The prevalence rates and 95% confidence intervals (CI) for falling and vestibular problems in the modified Romberg test were calculated. The associations between falling or vestibular problems in the modified Romberg test and possible associated factors were evaluated using the chi-square test and Student’s *t*-test in the univariate analysis. A multivariate logistic regression analysis with complex sampling was used to calculate adjusted odds ratios (ORs) and 95% CIs for falling or vestibular problems in the modified Romberg test according to each variable. Clinically important variables with a *p*-value < 0.05 were selected for the multivariate analysis using a logistic regression model. Interactions among sex, osteoporosis, and 25-OH vitamin D3 were estimated using linear regression analysis. All analyses were weighted to the Korean 2009 and 2010 standard population, which reflected weights to response rates, weights to sampling, and weights to the population structure of the KNHANES parent study. All *p*-values were two-sided, and *p*-values < 0.05 were considered significant. Several logistic models were accessed to search the best-matched model for multicollinearity. Variables with multicollinearity problems (Dual Energy X-ray Absorptiometry T score (DEXA T) score for total femur, lumbar, and femur neck) were not included in the logistic regression model (Table S1).

## 3. Results

### 3.1. Factors Associated with Falling and Vestibular Problems in the Modified Romberg Test

Various factors, including age, BMI, hearing loss, falling, limitation of activity, stress, EQ-5D index, visual disturbances, osteoarthritis, hypertension, diabetes, anemia, and osteoporosis had significant differences in terms of vestibular problems in the modified Romberg test group and control group in the univariate analysis (Table 1 and Table 2). Therefore, it was a prerequisite to adjust these factors before analyzing the relationship between bone metabolism and vestibular problems in the modified Romberg test (Table 2). Several bone metabolism parameters were significantly different according to the presence of vestibular problems in the modified Romberg test. Laboratory markers, including ALP (*p* < 0.001), PTH (*p* = 0.047) and 25-OH vitamin D3 (*p* < 0.001) levels, were significantly different depending on the presence of a vestibular problems in the modified Romberg test in the univariate analysis. Serum 25-OH vitamin D3 levels were significantly lower but ALP and PTH levels were higher in vestibular problems in the modified Romberg test group than those in the no-vestibular problems in the modified Romberg test group, with serum 25-OH vitamin D3 level showing a significant difference in the multivariate analysis (*p* < 0.001, OR = 0.951, 95% CI = 0.926–0.976). To exclude the collinearity issue between vitamin D levels and activity limitation, we analyzed the correlation between these factors. However, no correlation was observed (*p* = 0.54, chi-square).

Next, we analyzed factors associated with falling (Table 3). Several variable, including age, sex, BMI, tinnitus, hearing loss, limitation of activity, stress, EQ-5D index, visual disturbance, osteoarthritis, depressive mood, hypertension, diabetes, anemia, and osteoporosis were positively associated with falling in the univariate analysis (Table 3). Serum 25-OH vitamin D3, ALP, or PTH levels were not significantly different between falling and no-falling groups. After adjusting for possible confounding factors, osteoporosis was not related to falling in the multivariate analysis. Rather, tinnitus, limitation of activity, stress, EQ-5D index, and having hypertension as a comorbidity were associated with falling.

### 3.2. Sex Differences in Factors Associated with Vestibular Problems in the Modified Romberg Test

Because many factors related to bone metabolism were significantly related with vestibular problems in the modified Romberg test in the univariate analysis, as well as serum 25-OH vitamin D3 levels being significantly lower in vestibular problems in the modified Romberg test group than those in the no-vestibular problems in the modified Romberg test group in the multivariate analysis, we hypothesized that bone metabolism may affect vestibular problems in the modified Romberg test. Because bone metabolism in postmenopausal women is influenced by changes in estrogen levels [15], there may be differences between men and women in the degree of osteoporotic changes and related parameters. The interaction among vitamin D, sex, and osteoporosis were examined (Table 4). As results, sex was interacted with osteoporosis and mediated the associations with vestibular problems in the modified Romberg test (R Square = 0.012, *p* < 0.001). In addition, we conducted a subgroup analysis according to sex for the factors associated with vestibular problems in the modified Romberg test (Table S2). In males, hearing loss, falling, limitation of activity, quality of life in the EQ-5D index, osteoarthritis, anemia, and osteoporosis were associated with vestibular problems in the modified Romberg test in the univariate analysis. In females, hearing loss, falling, limitation of activity, quality of life in the EQ-5D index, visual disturbances, hypertension, diabetes, menopause, and osteoporosis were related with vestibular problems in the modified Romberg test in the univariate analysis.

**Figure 2:**
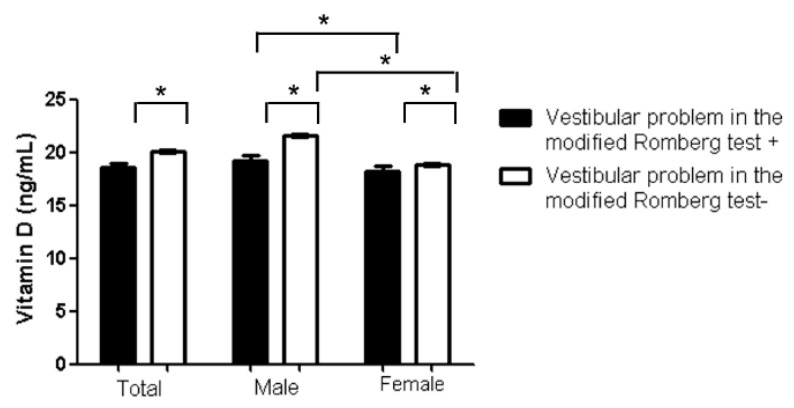
Serum vitamin D levels according to the vestibular problems in the modified Romberg test groups (* *p* < 0.05).

**Figure 3:**
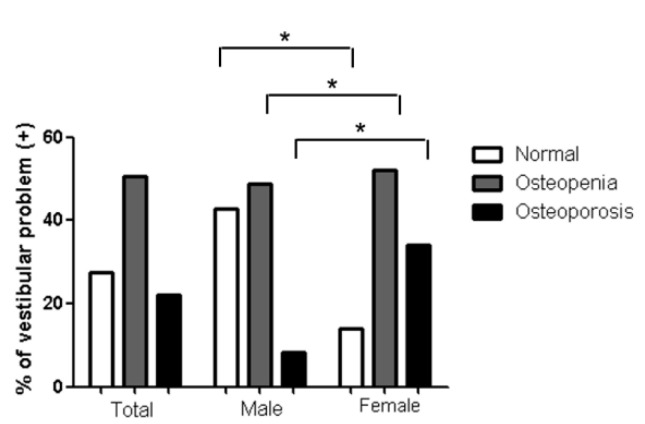
Prevalence of osteoporosis in the vestibular problems in the modified Romberg test in each group (* *p* < 0.05).

All DEXA T-scores of the total femur and femoral neck, as well as diagnoses of osteoporosis or osteopenia based on the lowest DEXA T-score, were related to vestibular problems in the modified Romberg test, in both male and female subgroups in the univariate analysis (Table S2). Serum vitamin D levels were associated with vestibular problems in the modified Romberg test for the male subgroup in the univariate analysis (*p* = 0.009). However, none of these factors were correlated in the multivariate analysis. The serum vitamin D levels were lower in females than that in males (*p* < 0.001) (Figure 2). In addition, the rate of osteoporosis in the participants with vestibular problems in the modified Romberg test was higher and in females than that in males (*p* < 0.001) (Figure 3). Interestingly, the women showed a significantly increased prevalence of vestibular problems in the modified Romberg test for the post-menopause group (*p* = 0.019). In the menopausal group of women, variables related to osteoporotic changes, including osteoporosis and DEXA T scores in total femur, femur neck, and lumbar regions, were related to vestibular problems in the modified Romberg test (Table S3). In addition, histories of hormone replacement therapy was lower in vestibular problems in the modified Romberg test group than the no-vestibular problems in the modified Romberg test group (9.5% vs. 18.1%, *p* = 0.035).

## 4. Discussion

In the present study, we identified several bone metabolism parameters that were significantly associated with vestibular problems in the modified Romberg test. Moreover, we demonstrated sex differences in bone metabolism parameters associated with vestibular problems in the modified Romberg test. The presence of menopause and a history of hormone replacement therapy were associated with vestibular problems in the modified Romberg test in females.

The association between vestibular dysfunction and osteoporosis has been proposed in several previous studies, which mostly involved small study populations [13,26]. A case-control study found higher postural sway and different balance control strategies using the hips for maintaining balance in computerized dynamic posturography in the osteoporosis group (*n* = 16) compared to the control group (*n* = 5) [13]. Another case-control study showed a higher rate of abnormal results in the stepping tandem gait test and subjective visual vertical test as well as the absence of ocular vestibular evoked myogenic potential in the osteoporosis (*n* = 11) group versus the control group (*n* =12) [26]. Two previous studies reported the association of osteoporosis with an increase prevalence of vestibular problems in the modified Romberg test in national cohort populations [9,11]. However, variables related to bone metabolism, including serum levels of vitamin D, PTH, and ALP, were not investigated in prior studies [9,11]. Moreover, physical mobility and visual function, which are factors that could influence balance, were not adjusted for in those studies. In the present study, indicators of osteoporotic changes, DEXA T-scores, and serum levels of vitamin D, PTH, and ALP were considered, and confounders such as physical activity and visual disturbance, were adjusted. Although many bone metabolism variables showed tendencies toward increased osteoporotic changes in falling and vestibular problems in the modified Romberg test groups compared to the no-vestibular problems in the modified Romberg test groups or no-falling groups after univariate analyses, no osteoporotic change variables were significantly associated with falling. However, decreased vitamin D levels were related to vestibular problems in the modified Romberg test.

**Figure 4:**
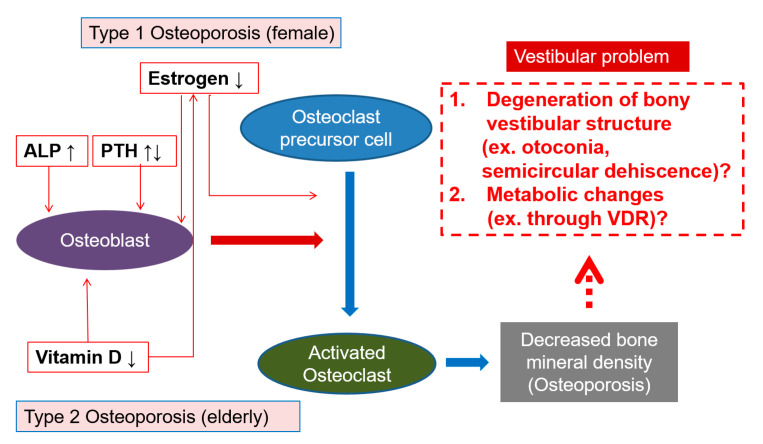
Schematic mechanisms underlying the relationship between bone metabolism and vestibular problem. Decreased estrogen levels in postmenopausal women disinhibit osteoclast activation and decrease activation of osteoblasts, resulting in bone resorption and subsequent osteoporotic changes (type 1 osteoporosis). The increase in parathyroid hormone due to secondary hypoparathyroidism induces specific cytokines that activate osteoclast precursor cells and induce osteoporotic changes (type 2 osteoporosis). Vitamin D has anabolic action in bone via activation of osteoblasts. The decrease in serum vitamin D levels can affect vestibular problems directly via dysfunction of the vitamin D receptor, which is expressed in vestibular organs (1), or by inducing osteoporotic changes related to degenerative changes in the otoliths and other vestibular organs (2).

Several mechanisms may explain why serum vitamin D levels influence the balance system (Figure 4), with decreased bone mineral density being the link between vitamin D deficiency and vestibular problems. A previous clinical study found utricular pathologies using a subjective visual vertical test [26]. In addition, an experimental study in osteoporotic rats found decreased density and morphologic changes of their otoconia [10]. Because otolith organs are composed of calcium carbonate, the absorption of calcium from otolith organs and the vestibular labyrinth could be affected in osteoporotic conditions due to reduced calcium reservoirs from intestinal absorption [27]. The absorption of calcium might also perturb the maintenance of low calcium levels of endolymph (280 μM) compared to perilymph (1 mM), which is essential for transduction of acceleration for maintaining balance in the vestibular labyrinth [28]. In addition, molecules related with the calcium transport system such as epithelial calcium channels, calcium buffer proteins of calbindins, sodium-calcium exchangers, and plasma membrane calcium-ATPases, are expressed in the epithelia of the semicircular canal duct, with their functions possibly being influenced by osteoporotic changes [29].

Besides decreased bone mineral density, vitamin D could affect vestibular function via vitamin D-related signal pathways. The hormonally active form of vitamin D3, dihydroxyvitamin D3, binds to nuclear vitamin D receptors (VDR) [30]. Numerous neurons and glial cells express VDR [31]. Several studies have shown a significant role of vitamin D in the vestibular system and maintenance of balance. A previous clinical trial study reported that the intake of vitamin D and its metabolites of calcitriol decreased the incidence of falling and vertebral fractures in the elderly [32]. An animal study showed that a mutation in VDR accelerates neural degeneration in the cochlea [33]. Moreover, VDR is expressed in the saccule, utricle, and semicircular canal duct [34], with VDR deficiency found to induce vertigo [35,36]. This suggests that otovestibular physiology may be influenced by vitamin D. We found no correlation between falling and vitamin D levels. This may have been related to the heterogeneity of the causes of falling in the falling group, which included impaired balance, anxiety, debilitated motor function, and visual impairment.

The relationship of variables influencing bone metabolism with vestibular problems in the modified Romberg test was different according to sex in this study. In addition, hormone replacement therapy was negatively correlated with vestibular problems in the modified Romberg test in postmenopausal women. Estrogen levels are low in postmenopausal women. This hormonal change subsequently drives changes in bone metabolism toward osteoclastic bone lysis [15]. Thus, osteoporosis is more common in postmenopausal women than in men of the same age [16]. One study showed that BPPV tends to occur more frequently in postmenopausal women than in men [37]. From these findings, we hypothesized that there are different pathophysiological factors related to estrogen in women. To validate this hypothesis, we conducted a subgroup analysis based on sex, and demonstrated that osteoporosis and estrogen levels are factors associated with vestibular problem in females. Estrogens are bone anabolic hormones which prevent activation of osteoclast precursor cells. Estrogen deficiency in postmenopausal women results in the upregulation of several cytokines and accelerated bone resorption [38] (Figure 4). Moreover, one study demonstrated the presence of estrogen-related receptor mRNA in the vestibular ganglion [39], suggesting a role for estrogens in the vestibular system.

Vitamin D status in this study was measured using the nominal gold standard method. We included several variables influencing bone metabolism and the balance system. In particular, because vitamin D is synthesized by sunlight exposure in humans [40], limitations in activity, which is another factor significantly associated with vestibular problems, can result in insufficient sunlight exposure and decreased vitamin D levels. To exclude this confounding factor, we included activity limitation as a possible factor associated with vestibular problems. However, when we analyzed the relationship between vitamin D levels and limitations in activity, no correlation was found. Therefore, serum vitamin D level is an independent factor associated with vestibular problems regardless of sun exposure. Moreover, many possible confounding factors, including BMI, and other underlying diseases, were adjusted for in the present study.

One limitation in the study was its cross-sectional design, which led to restricted evaluation of the causal relationship between vestibular problems and bone metabolism. For example, vestibular problems and osteoporosis would reciprocally increase the risk of other diseases. Vestibular problems can also cause falling, potentially resulting in fractures and increased osteoporotic changes, while osteoporosis increases the risk of falling or vestibular problems. Therefore, we could not determine the causal relationship between osteoporotic changes and vestibular problems. To minimize this limitation, we used several variables related to bone metabolism and performed a subgroup analysis. Further studies elucidating the mechanisms underlying the effect of vitamin D levels and changes in bone metabolism on vestibular problems are therefore warranted. For the definition of vestibular problems in the modified Romberg test, we could not perform comprehensive vestibular function tests such as the caloric and rotary chair tests since this study was based on a large representative nationwide population cohort. Thus, the potential of misdiagnosis could not be completely excluded in this study. However, a modified Romberg test condition of four has been found to detect vestibular dysfunction with high test performance, since examinees are made to stand on memory foam with their eyes closed, forcing them to rely on their vestibular functions to maintain balance [41,42]. Lastly, the unconcerned confounders, such as inner ear diseases cannot be accessed in this study.

## 5. Conclusions

Osteoporotic changes in bone metabolism due to decreased serum vitamin D levels were associated with vestibular problems in the modified Romberg test as an independent factor with various covariates, including age and BMI. In addition, sex differences in bone metabolism were found, with postmenopausal women presenting with lower vitamin D levels compared to men. Moreover, hormone replacement therapy was negatively associated with vestibular problems in the modified Romberg test in menopausal women.

## Figures and Tables

**Table 1:** Prevalence of falling and vestibular problems in the modified Romberg test by sex, distribution of age and body mass index BMI in each group (*n* = 4054).

	Vestibular Problems in the Modified Romberg test (+) (*n* = 158)	Vestibular Problems in the Modified Romberg test (−) (*n* = 3896)	*p*-Value ^c^	OR	95% CI	Falling (+) (*n* = 626)	Falling (−) (*n* = 3428)	*p*-Value ^c^	OR	95% CI
Age	70.98 ± 8.19	62.55 ± 8.37	<0.001 *	1.097	1.067–1.127	65.32 ± 8.67	62.43 ± 8.42	0.012 *	1.018	1.004–1.032
Male	3.4 ^a^	96.6 ^a^	0.1182 ^b^	1.248	0.901–1.729	11.0	89.0	<0.001 *^,b^	1.645	1.307–2.071
Female	4.3 ^a^	95.7 ^a^				19.0	81.0			
BMI (kg/m^2^)	22.75 ± 3.13	24.07 ± 3.04	<0.001 *	0.863	0.809–0.920	23.76 ± 3.31	24.07 ± 3.00	0.004	0.952	0.921–0.985

Abbreviations: CI; confidence interval, BMI; body mass index, OR; odds ratio. ^a^ Percentage of the Korean population age 50 years or older. ^b^ Rao-Scott Chi-square test was used. ^c^ For the age-related trend test, logistic regression analysis was used. * *p* < 0.05.

**Table 2:** Analysis of factors potentially associated with “vestibular problems in the modified Romberg test” in participants over 50 years old.

	% ^a^	Vestibular Problems in the Modified Romberg Test (+)	Vestibular Problems in the Modified Romberg Test (−)	The Univariable Analysis			The Multivariable Analysis ^†^					
				*p*-Value	OR	95% CI	*p*-Value	OR		95% CI		
**Otolaryngologic conditions (physical examination and questionnaire)**												
**Tinnitus**												
No (%)	75.5	3.7	96.3	Referent								
Yes (%)	24.5	4.5	95.5	0.238	1.237	0.869–1.760						
Hearing loss												
No (%)	76.3	2.6	97.4	Referent			Referent					
Yes (%)	23.7	8.1	91.9	<0.001 *	3.337	2.421–4.599	0.010 *	1.63		1.123–2.365		
Falling												
No (%)	84.6	2.9	97.1	Referent								
Yes (%)	15.4	9.1	90.9	<0.001 *	3.3	2.357–4.621						
General conditions for activity												
Limitation of activity												
No (%)	78.6	2.7	97.3	Referent			Referent					
Yes (%)	21.4	8.4	91.6	<0.001 *	3.355	2.431–4.631	0.003 *	1.891		1.244–2.874		
Stress												
No (%)	75.6	3.5	96.5	Referent								
Yes (%)	24.4	5.1	94.9	0.031 *	1.46	1.036–2.057	0.451	1.169		0.779–1.753		
EQ-5D index (Mean) ^b,†^	0.91 ± 0.14	0.81 ± 0.21	0.91 ± 0.14	<0.001 *	0.046	0.022–0.096	0.11	0.404		0.133–1.226		
Visual disturbance												
No (%)	98.6	3.8	96.2	Referent			Referent					
Yes (%)	1.4	10.9	89.1	0.007 *	3.099	1.307–7.346	0.503	0.699		0.245–1.994		
Underlying diseases												
Diagnosis of stroke												
No (%)	97.1	3.9	96.1	Referent								
Yes (%)	2.9	5.1	94.9	0.5	1.334	0.577–3.080						
Diagnosis of osteoarthritis												
No (%)	78.9	3.4	79.3	Referent			Referent					
Yes (%)	21.1	5.7	94.3	0.002 *	1.718	1.216–2.428	0.343	1.223		0.807–1.851		
Depressive mood												
No (%)	83.3	3.7	96.3	Referent								
Yes (%)	16.7	4.7	95.3	0.23	1.275	0.857–1.897						
Diagnosis of depression												
No (%)	95	3.9	96.1	Referent								
Yes (%)	5	4	96	0.962	1.018	0.493–2.103						
Diagnosis of Hypertension												
No (%)	61.2	3	97	Referent			Referent					
Yes (%)	38.8	5.3	94.7	<0.001 *	1.839	1.337–2.530	0.115	1.349		0.929–1.958		
Diagnosis of Diabetes												
No (%)	86.1	3.6	96.4	Referent			Referent					
Yes (%)	13.9	5.7	94.3	0.019 *	1.613	1.083–2.403	0.18	1.35		0.870–2.095		
Diagnosis of Anemia												
No (%)	90.7	3.6	96.4	Referent			Referent					
Yes (%)	9.3	6.4	93.6	0.010 *	1.803	1.152–2.822	0.361	0.785		0.467–1.319		
Osteoporosis												
Normal	27.5	1.4	98.6				Referent					
Osteopenia	50.5	4	96	<0.001 *	2.953	1.659–5.256	0.15	1.555		0.853–2.835		
Osteoporosis	22	6.8	93.2	<0.001 *	5.205	2.873–9.430	0.43	1.303		0.675–2.513		
DEXA T score (total femur)	−0.35 ± 1.03	−0.97 ± 1.14	−0.32 ± 1.01	<0.001 *	0.531	0.452–0.624						
DEXA T score (femur neck)	−1.24 ± 1.06	−1.89 ± 1.15	−1.21±1.05	<0.001 *	0.523	0.444–0.615						
DEXA T score (lumbar)	−1.22 ± 1.36	−1.64 ± 1.49	−1.20 ± 1.35	<0.001 *	0.776	0.683–0.882						
vitamin D (ng/mL)	20.05 ± 7.22	18.57 ± 6.78	20.11 ± 7.23	<0.001 *	0.969	0.946–0.992	<0.001 *	0.951		0.926–0.976		
alkaline phosphatase (IU/L)	247.18 ± 75.81	270.26 ± 94.72	246.24 ± 74.81	<0.001 *	1.003	1.002–1.005	0.108	1.002		1.000–1.004		
PTH (pg/mL)	68.89 ± 29.99	73.59 ± 32.58	68.70 ± 29.86	0.047 *	1.004	1.000–1.008						

Some factors showing statistical significance in univariable analysis, such as DEXA T score at various anatomic sites, were not included in the logistic regression model due to the multicollinearity problems. Abbreviations: OR, odds ratio; CI, confidence interval; BMI, body mass index. ^a^ Sample weights applied. ^b^ Continuous variables are presented as mean ± standard deviation. ^†^ Clinically important variables with *p* values < 0.05 in the univariable analysis (age, sex, hearing loss, limitation of activity, EQ-5D index, visual disturbance, osteoarthritis, depression, hypertension, diabetes, anemia, osteoporosis, serum vitamin D level, alkaline phosphatase, and parathyroid hormone (PTH)) were included in the multivariable analysis. * *p* < 0.05

**Table 3:** Analysis of factors potentially associated with “Falling” in participants over 50 years old.

	% ^a^	Falling (+)	Falling (−)	The Univariable Analysis			The Multivariable Analysis ^†^					
				*p*-Value	OR	95% CI	*p*-Value		OR	95% CI		
**Otolaryngologic conditions (Physical examination and questionnaire)**												
**Tinnitus**												
No (%)	75.5	24.5	75.5	Referent			Referent					
Yes (%)	24.5	12.5	87.5	<0.001 *	2.281	1.905–2.730	<0.001 *		1.907	1.562–2.328		
Hearing loss												
No (%)	76.3	13.7	86.3	Referent			Referent					
Yes (%)	23.7	21.0	79.0	<0.001 *	1.666	1.383–2.007	0.152		1.176	0.942–1.468		
General conditions for activity												
Limitation of activity												
No (%)	78.6	12.6	81.3	Referent			Referent					
Yes (%)	21.4	26.1	73.9	<0.001 *	2.457	2.043–2.954	0.002 *		1.451	1.145–1.840		
Stress												
No (%)	75.6	13.0	87.0	Referent			Referent					
Yes (%)	24.4	22.5	77.5	<0.001 *	1.937	1.614–2.326	0.004 *		1.391	1.110–1.743		
EQ-5D index (Mean) ^†^	0.90 ± 0.15 ^b^	0.84 ± 0.19 ^b^	0.92±0.13 ^b^	<0.001 *	0.051	0.030–0.086	<0.001*		0.286	0.141–0.578		
Visual disturbance												
No (%)	98.6	15.2	84.8	Referent								
Yes (%)	1.4	36.4	63.6	<0.001 *	3.199	1.835–5.580	0.373		1.342	0.702–2.566		
Underlying diseases												
Diagnosis of Stroke												
No (%)	97.1	15.3	84.7	Referent								
Yes (%)	2.9	21.2	78.8	0.082	1.492	0.951–2.339						
Diagnosis of Osteoarthritis												
No (%)	78.9	13.9	86.1	Referent			Referent					
Yes (%)	21.1	21.4	78.6	<0.001 *	1.688	1.393–2.045	0.707		1.046	0.827–1.324		
Depressive mood												
No (%)	83.3	13.9	86.1	Referent			Referent					
Yes (%)	16.7	23.0	77.0	<0.001 *	1.844	1.504–2.260	0.337		1.132	0.879–1.459		
Diagnosis of Hypertension												
No (%)	61.2	13.6	86.4	Referent								
Yes (%)	38.8	18.3	81.7	<0.001 *	1.425	1.200–1.691	0.045 *		1.230	1.005–1.504		
Diagnosis of Diabetes												
No (%)	86.1	15.0	85.0	Referent			Referent					
Yes (%)	13.9	18.3	81.7	0.042 *	1.274	1.009–1.608	0.423		1.112	0.857–1.443		
Diagnosis of Anemia												
No (%)	90.7	14.8	85.2	Referent			Referent					
Yes (%)	9.3	21.5	78.5	0.001 *	1.578	1.214–2.052	0.910		1.018	0.747–1.387		
Osteoporosis												
Normal	27.5	10.4	89.6	Referent								
Osteopenia	50.5	15.5	84.5	<0.001 *	1.582	1.247–2.007	0.552		1.083	0.833–1.407		
Osteoporosis	22.0	21.9	78.1	<0.001 *	2.422	1.864–3.147	0.574		1.099	0.790–1.529		
DEXA T score (total femur)	−0.35 ± 1.03 ^b^	−0.59 ± 1.06 ^b^	−0.30 ± 1.01 ^b^	<0.001 *	0.760	0.699–0.828						
DEXA T score (femur neck)	−1.24 ± 1.06 ^b^	−1.56 ± 1.07 ^b^	−1.18 ± 1.05 ^b^	<0.001 *	0.706	0.649–0.769						
DEXA T score (lumbar)	−1.22 ± 1.36 ^b^	−1.52 ± 1.38 ^b^	−1.16 ± 1.35 ^b^	<0.001 *	0.818	0.766–0.875						
vitamin D (ng/mL)	20.05 ± 7.22 ^b^	19.79 ± 6.89 ^b^	20.10 ± 7.28 ^b^	0.329	0.994	0.982–1.006						
alkaline phosphatase (IU/L)	247.18 ± 75.81 ^b^	251.01 ± 79.11 ^b^	246.48 ± 75.19 ^b^	0.169	1.001	1.000–1.002						
PTH (pg/mL)	68.89 ± 29.99 ^b^	67.66 ± 29.27 ^b^	69.11 ± 30.11 ^b^	0.266	0.998	0.995–1.001						

Some factors showing statistical significance in univariable analysis, such as DEXA T score at various anatomic sites, were not included in the logistic regression model due to the multicollinearity problems. Abbreviations: OR, odds ratio; CI, confidence interval; BMI, body mass index. ^a^ Sample weights applied. ^b^ Continuous variables are presented as mean ± standard deviation. ^†^ Clinically important variables with *p* values < 0.05 in the univariable analysis (age, sex, tinnitus, hearing loss, limitation of activity, stress, EQ-5D index, visual disturbance, stroke, osteoarthritis, depressive mood, hypertension, diabetes, anemia, and osteoporosis) were included in the multivariable analysis. * *p* < 0.05

**Table 4:** Interactions among vitamin D, sex, and osteoporosis for the relation with the vestibular problems in the modified Romberg test.

Model	R	R Square	F	*p*
Vitamin D	0.052	0.003	9.929	0.002 *
Vitamin D * Sex	0.057	0.003	5.909	0.003 *
Vitamin D * Sex * Osteoporosis	0.108	0.012	14.374	<0.001 *

* *p* < 0.05.

## Supplementary Materials

The following are available online at https://www.mdpi.com/2077-0383/9/8/2415/s1, Table S1: Linear regression analysis for multicollinearity on osteoporosis, Table S2: Subgroup analysis of associate factors of vestibular problems in the modified Romberg test between male and female, Table S3: Subgroup analysis of associate factors of vestibular problems in in the modified Romberg test in menopause women (*n* = 2103).
