# Supplementary material for: Association between Bone Metabolism and Vestibular Problems in the Modified Romberg Test: Data from the 2009–2010 Korean National Health and Nutrition Examination Survey
Source: J Clin Med. 2020 Jul 28;9(8):2415. doi: 10.3390/jcm9082415 (PMC7466128; doi:10.3390/jcm9082415)
Supplement: Supplementary file 1 [file jcm-09-02415-s001.pdf]

Table S1. Linear regression analysis for multicollinearity on osteoporosis

Dependent variable	Independent variable	B	β	t	*p*	VIF†
**Osteoporosis**	DEXA T score (total femur)	-.027	-.038	-2.071	.038*	4.852
	DEXA T score (lumbar)	-.259	-.500	-41.530	<0.001*	2.070
	DEXA T score (femur neck)	-.265	-.398	-21.478	<0.001*	4.900
	vitamin D (ng/mL)	-.001	-.014	-1.692	.091	1.046
	alkaline phosphatase (IU/L)	4.368E-05	.005	.550	.583	1.062
	PTH (pg/ mL)	.000	-.010	-1.156	.248	1.059

* $p < 0.05$

†VIF: variance inflation factor

Table S2. Subgroup analysis of associate factors of vestibular problems in the modified Romberg test between male and female.

Variables	Male (n = 1,801)				Female (n =2.253)			
	%[a]	Vestibular problems in the modified Romberg test (+) (n = 62)	Vestibular problems in the modified Romberg test (-) (n = 1,739)	*P* Value	%[a]	Vestibular problems in the modified Romberg test (+) (n = 96)	Vestibular problems in the modified Romberg test (-) (2,157)	*P* Value
Otolologic conditions								
Tinnitus								
No (%)	75.7	3.1	96.9	0.092	75.3	4.2	95.8	0.809
Yes (%)	24.3	4.6	95.4		24.7	4.5	95.5	
Hearing loss								
No (%)	72.6	1.8	98.2	<.001*	79.3	3.2	96.8	<.001*
Yes (%)	27.4	7.9	92.1		20.7	8.4	91.6	
Falling								
No (%)	89	2.8	97.2	<0.001*	81	3.1	96.9	<.001*
Yes (%)	11	8.5	91.5		19	9.4	90.6	
General conditions for activity								
Limitation of activity								
No (%)	83	2.3	97.7	<.001*	75.1	3	97	<.001*

Yes (%)	17	8.8	91.2		24.9	8.2	91.8	
EQ-5D index†	0.94±0.12	0.84±0.21	0.94±0.11	<.001*	0.88±0.16	0.79±0.21	0.88±0.15	<.001*
Visual disturbance								
No (%)	98.5	3.4	96.6	0.238	98.8	4.1	95.9	0.029*
Yes (%)	1.5	7.4	92.6		1.2	14.3	85.7	
Underlying diseases								
Stroke								
No (%)	96.4	3.5	96.5	0.611	97.6	4.2	95.8	0.284
Yes (%)	3.6	3.1	96.9		2.4	7.5	92.5	
Osteoarthritis								
No (%)	91.8	3.1	96.9	0.014*	68.5	3.8	96.2	0.092
Yes (%)	8.2	7.5	92.5		31.5	5.4	94.6	
Depression								
No (%)	97.9	3.5	96.5	0.639	92.7	4.2	95.8	0.686
Yes (%)	2.1	0	100		7.3	4.9	95.1	
Hypertension								
No (%)	62	2.9	97.1	0.109	60.6	3.1	96.9	0.001*
Yes (%)	38	4.4	95.6		39.4	6.1	93.9	

Diabetes								
No (%)	84.7	3.4	96.6	0.857	87.3	3.8	96.2	0.004*
Yes (%)	15.3	3.6	96.4		12.7	7.7	92.3	
Anemia								
No (%)	92.9	3.1	96.9	0.01*	89	4.1	95.9	0.244
Yes (%)	7.1	7.8	92.2		11	5.6	94.4	
Menopause								
No (%)	NA				6.7	0.7	99.3	0.019*
Yes (%)					93.3	4.5	95.5	
Osteoporosis								
Osteoporosis								
Normal	42.8	1.6	98.4	0.002*	13.9	0.7	99.3	<.001*
Osteopenia	48.8	4.8	95.2		52.1	3.2	96.8	
Osteoporosis	8.4	3.4	96.6		34	7.5	92.5	
DEXA T score (total femur)	-0.10±0.930	-0.53±1.086	-0.09±0.920	<0.001*	-0.55±1.06	-1.25±1.10	-0.51±1.04	<.001*
DEXA T score (femur neck)	-0.82±0.950	-1.31±1.087	-0.80±0.941	<0.001*	-1.58±1.02	-2.26±0.97	-1.55±1.02	<.001*

DEXA T score (lumbar)	-0.70±1.298	-0.83±1.501	-0.70±1.290	0.457	-1.63±1.27	-2.20±1.20	-1.61±1.27	<.001*
vitamin D (ng/mL)	21.53±7.323	19.15±6.239	21.62±7.346	0.009*	18.86±6.91	18.20±7.11	18.89±6.90	0.337
alkaline phosphatase (IU/L)	240.99±71.663	273.95±84.657	239.81±70.902	<0.001*	252.13±78.64	267.88±101.05	251.42±77.45	0.045*
PTH (pg/ mL)	67.05±25.865	73.10±28.369	66.83±25.754	0.061	70.36±32.84	73.91±35.18	70.20±32.73	0.279

Some factors showing statistical significance in univariable analysis, such as DEXA T score at various anatomic sites, were not included in the logistic regression model due to the multicollinearity problems.

[a] Sample weights applied. [b] Continuous variables are presented as mean ± standard deviation.

[†] Clinically important variables with *P* values < 0.05 in the univariable analysis were included in the multivariable analysis.

*$p < 0.05$

Table S3. Subgroup analysis of associate factors of vestibular problems in in the modified Romberg test in menopause women (n = 2,103)

Variables	Total	Vestibular problems in the modified Romberg test (+) (n = 95)	Vestibular problems in the modified Romberg test (-) (n = 2,008)	*P* Value	OR	95% CI
Hormone replacement therapy (%)						
Yes	17.7	9.5	90.5	0.035*	0.473	0.236-0.948
No	82.3	18.1	81.9			
Osteoporosis (%)						
Normal	13.9	0.7	99.3			
Osteopenia	52.1	3.2	96.8	0.039*	4.515	1.077-18.937
Osteoporosis	34.0	7.5	92.5	0.001*	10.971	2.650-45.414
DEXA T score (total femur)	-0.62±1.02	-1.28±1.07	-0.59±1.01	<.001*	0.497	0.400-0.616
DEXA T score (femur neck)	-1.65±0.98	-2.28±0.96	-1.62±0.98	<.001*	0.472	0.373-0.597
DEXA T score (lumbar)	-1.74±1.21	-2.23±1.18	-1.72±1.21	<.001*	0.682	0.562-0.827
vitamin D (ng/mL)	19.01±6.98	18.17±7.14	19.05±6.977	0.227	0.981	0.951-1.012
alkaline phosphatase (IU/L)	255.80±79.05	268.92±101.07	255.18±77.83	0.097	1.002	1.000-1.004
PTH (pg/ mL)	70.46±33.37	74.08±35.33	70.29±33.27	0.280	1.003	0.998-1.007

$^*p < 0.05$
